# Assessing the added value of context during stress detection from wearable data

**DOI:** 10.1186/s12911-022-02010-5

**Published:** 2022-10-15

**Authors:** Marija Stojchevska, Bram Steenwinckel, Jonas Van Der Donckt, Mathias De Brouwer, Annelies Goris, Filip De Turck, Sofie Van Hoecke, Femke Ongenae

**Affiliations:** 1 IDLab, Ghent University - imec, Technologiepark Zwijnaarde 126, Ghent, Belgium; 2 OnePlanet Research Center, imec, Bronland 10, 6708 Wageningen, The Netherlands

**Keywords:** Context-aware, Machine learning, Wearable health, Stress

## Abstract

**Background:**

Insomnia, eating disorders, heart problems and even strokes are just some of the illnesses that reveal the negative impact of stress overload on health and well-being. Early detection of stress is therefore of utmost importance. Whereas the gold-standard for detecting stress is by means of questionnaires, more recent work uses wearable sensors to find continuous and qualitative physical markers of stress. As some physiological stress responses, e.g. increased heart rate or sweating and chills, might also occur when doing sports, a more profound approach is needed for stress detection than purely considering physiological data.

**Methods:**

In this paper, we analyse the added value of context information during stress detection from wearable data. We do so by comparing the performance of models trained purely on physiological data and models trained on physiological and context data. We consider the user’s activity and hours of sleep as context information, where we compare the influence of user-given context versus machine learning derived context.

**Results:**

Context-aware models reach higher accuracy and lower standard deviations in comparison to the baseline (physiological) models. We also observe higher accuracy and improved weighted F1 score when incorporating machine learning predicted, instead of user-given, activities as context information.

**Conclusions:**

In this paper we show that considering context information when performing stress detection from wearables leads to better performance. We also show that it is possible to move away from human labeling and rely only on the wearables for both physiology and context.

**Supplementary Information:**

The online version contains supplementary material available at 10.1186/s12911-022-02010-5.

## Introduction

Stress is defined by Selye [1] as “the non-specific response of the body to any demand for change, and is the most frequent work-related health problem in Europe [2]. Today, 80% of the people experience stress at work, which often evolves in long-term stress disorders, including burn-out, heart disease, cancer, lung disease, accidents, cirrhosis of the liver and suicide [3]. Besides the health-related issues, the European Union estimated a loss of 240 billion euros per year due to mental illnesses where stress is seen as one of the causes or instigators of most of these mental illnesses [4].

In the early stages, stress introduces sleeping difficulties, headaches, fatigue and gastrointestinal upsets. To timely identify stress, it is essential to detect and analyze these symptoms from early on. Current gold-standard methods use user-driven interviews and questionnaires, such as the Perceived Stress Questionnaire (PSQ). These reports have a limited predictive potential because patients often fill them in when their stress-related disorders have already reached a late stage [5].

When experiencing stress a number of bodily changes take place. This response consists of changes in several physiological biosignals such as skin temperature (ST, which is the temperature on the skin surface that touches the sensor and not the body core), electrodermal activity (EDA, also known as Galvanic Skin Response (GSR), which is an indicator of sweating), heart rate (HR), blood volume pressure (BVP), among others [6].

In recent years, the use of smartphones and wearable devices such as chest heart rate monitors and wrist worn bands or smartwatches has increased and many people own one or several such devices [7, 8]. Most of these devices (or a combination thereof) can measure several of the physiological biosignals mentioned. As a consequence, more recent works use smartphones and wearable heart rate monitors to assess stress and detect stress-induced changes in the users’ daily lives [9, 10]. By exploiting these smart wearable devices and advanced computing algorithms, researchers have come up with several automatic stress monitoring schemes to determine and detect stress in a wide variety of situations [11]. Since these wearable devices are most often non-obtrusive, they introduce a great opportunity for long term and real-life stress monitoring.

However, some changes in physiological biosignals are not necessarily the result of experiencing stress. For example increased HR and change in ST might also occur during a physical exercise [12, 13]. The current stress detection tools do not take into account this context information, and therefore make incorrect stress predictions for these cases, resulting in a less reliable stress detection strategy. Motion artifacts in GSR and Photoplethysmogram (PPG, the sensor typically embedded in wrist-worn wearable devices from which the HR is derived) additionally distort these signals and make them unreliable in situations with increased activity [10].

To cope with these situations, research should go beyond the physiological biosignals and consider context information. Context information can help mitigate the problems mentioned here above: having the information on current or recent physical activities allows for a more correct interpretation of the physical biosignals and avoids certain activities being misinterpreted as stress. Getting to know the activities can be done through (daily/hourly) questionnaires and/or Ecological Momentary Assessments (EMAs, which are questions to the user at strategically chosen moments). However, traditional questionnaires suffer from recall bias [14] and too sparse data if timed infrequently, and also increase the user burden and the drop-out (ignoring the questions) rate if asked too frequently. Even though the EMAs may reduce some of these problems, they may still not be sustainable in long-term monitoring, especially if not implemented properly [15, 16]. When aiming for longitudinal and real-world studies on understanding stress and its consequences, we therefore need to move away from user-provided context information and shift towards obtaining this information automatically. Besides relieving the user from the burden of self-reporting, label quality this way also shifts from subjective to objective labels. As smartphones and wearable devices are equipped with many sensors, this sensor data can be used to infer some of this activity information automatically. The accelerometer signal in both the smartphone and the wearable device is adequate for detecting the person’s activity and sleep period [17, 18].

Besides using context information for correct interpretation or adequate use of the biosignals, and therefore possibly reducing false positives, it can also give a prior probability or indicate increased chances of a person experiencing stress. It is for example known that stress and sleep deprivation are linked, sometimes even leading to a vicious circle, where lack of sleep causes stress and anxieties, which in turn lead to insomnia [19]. Therefore, we can expect that a person feels irritable and stressed more after (several) nights of short sleep. In a similar way as before, this context information can be obtained by explicitly asking the user, or it can be automatically derived using smartphone and/or wearable device sensors.

This research is two-fold. First, we assess the added value of using context data for stress detection. We do so by considering two context modalities: (a) the activity performed by the user and (b) the user’s sleeping behavior. We compare the accuracy obtained using (a) only physiological features and (b) physiological and context features. Second, we automate the activity and sleep detection and perform the same experiments, this time incorporating the automatically derived context features instead of the user-given ones. We additionally compare the results obtained using user-provided versus automatically derived context features.

The rest of the paper is organized as follows: "Related work" section discusses the related work regarding stress assessment using wearable devices in various environments. In "Dataset" section discusses datasets used for this research. The activity recognition and sleep detection modules, which are used to automatically derive contextual information, are described in "Automated context retrieval" section. In "Experiments and results" section describes the two performed experiments to assess the impact of context on stress detection and the according results. A detailed discussion of the results and final remarks of this research are given in "Discussion" and "Conclusion" sections respectively.

## Related work

Multiple stress and wearable-related survey papers describe the different types of sensors, machine learning (ML) techniques and the various purposes for which the wearables were used [10, 20–24].

In this section, we briefly summarize the existing stress detection studies and their methodologies. Studies that use data-driven ML techniques come in two broad groups based on a defined (sub)goal and duration. The first group, in which the studies are of short duration, aims at acute stress detection. In the second group, studies are longitudinal, and their goal is to identify chronic stress.

### Short-term or acute stress detection

The duration of short-term stress detection studies ranges from several minutes up to several days. The experiments in these studies are always performed in a controlled lab environment and often try to tackle a concrete problem within a specific domain. Holmgard et al. performed a stress detection experiment to investigate Post Traumatic Stress Disorder (PTSD) via a computer game [25]. A wearable device which measures the skin conductance was used to find (unsupervised) correlations between physiological responses and the subjective stress evaluations. More recently, heart rate sensors are being used to capture PTSD in a supervised manner [26].

In the automobile domain, Keshan et al. applied ML methods to detect stress under different driving conditions [27]. The heart rate signals were derived using an electrocardiogram (ECG). This work was later on improved and used in diverse frameworks to capture stress levels while driving [28, 29]. However, ECG is not an ideal sensor for daily use and non-intrusive stress monitoring. Even with wearable sensors such as the Apple Watch, that have ECG sensors embedded, proper analysis requires the person to be resting and place the arm with the watch flat on a table or lap while touching the watch with the finger of the opposite hand. As a result, this is impossible to perform during driving, or even during any other activity, without interruption and is thus a major hindrance for adopting these sensors for real-life applications of stress detection.

More recently, physiological features that could be used as predictors of stressful activities and states of anxiety were examined in academic environments using an Arduino board and 5 low-cost sensors. The stress due to short activities performed by 21 students could be identified with an high accuracy using data from heart rate, skin temperature and oximetry signals and four derived physiological features.

Video-based interrogations are also frequently used to detect or analyze stressful events. Carneiro et al. experimented with such an interrogation framework to capture human-behavioral stress through cameras and touch responsiveness on a tablet or smartphone. They showed that each user is affected by stress in a specific way [30]. Giakoumis et al. adopted a similar approach to detect stress by using the Stroop color word test [31] in their experiments. In this test, the name of a color is printed in a color that mismatches its meaning (i.e., the term “red” printed in blue ink instead of red ink) [32]. The participants were asked to name the color of the word. This task takes longer than usual and induces stress. The wearable sensors, as well as the camera, could capture this stress event. In later studies, the use of thermal cameras increased the performance of these video-based stress detection studies [33]. Similar to ECG is the potential of using cameras for daily stress monitoring rather limited.

Besides video, short-term stress studies can also use sound to detect short-term stress in controlled environments. Lu et al. used a smartphone’s microphone to capture data in 3 situations: (1) an indoor job interview, (2) performing a competitive marketing job, (3) a neutral task [34]. They used GSR sensor readings as the ground truth, where an increase in the GSR values indicated stress. Later on, this “in-the-wild” speech recognition for stress detection is improved by using more advanced techniques, such as modulation spectral features and convolutional neural networks (CNN) [35]. While sound and speech-based stress detection looks promising, noise and external sounds make them impractical outside the lab environments.

More standardized tests are based on the Trier Social Stress Test (TSST) protocol [36], which includes both public speaking and cognitive tasks that place participants under high cognitive load. Mozos et al. use TSST, a variety of sensors and ML to classify stressful and neutral situations [37]. Their setup also indicated that the first generation wearable devices could only be useful in controlled, short-term tests and are not suitable for longitudinal monitoring in real-world settings. New studies show that adaptations of the TSST in and enterprise contexts together with wrist-worn wearables, can still be beneficial [38].

Acute stress detection research neglect or rarely take the user’s profile and contextual information into account. Most of the short-term experiments performed in a predefined lab setup hold good results within this environment. It is, in most cases, somewhat unrealistic that such lab environments resemble real-world situations or that cameras and microphones could be used in a non-intrusive way.

### Long-term or chronic stress detection

Long-term stress detection tests aim to capture real-life stress events. The experiments are, therefore, performed in open environments rather than controlled laboratory settings. Sano and Picard conducted a 5-day study in which the participants were asked at the beginning to fill in several questionnaires. They were then given a wrist-worn wearable and wore it for 5 days [39]. During these 5 days, every morning and evening, the participants filled in a survey in which they reported their sleeping behavior, daily mood and general stress level. Despite the limited number of participants and data, this study extracted stress associated features from the wearable sensor data and mobile phone usage.

Garcia-Ceja et al. used a similar smartphone setup to detect work-related stress [40]. The participants collected accelerometer data for eight weeks. Stress levels were registered three times a day, during their working hours, using their phones. Both a user-specific and global ML model were designed based on this data.

Muaremi et al. conducted a more extensive, four months study in which they collected audio, communication and physical activity data during the workday. They also collected heart rate variability data at night during the sleep period [9]. Four times a day, participants filled out the Positive and Negative Affect Schedule (PANAS) [41] questionnaire and provided a voice message in which they speak about their activity at that time.

Another longitudinal stress study is the SWEET study (Stress in the Work EnvironmEnT), a comprehensive, cross-sectional study on an office workers’ population of 1002 healthy volunteers, who were monitored continuously for five consecutive days. SWEET collected baseline psychological information together with five consecutive days of free-living physiological data through wearables. Participants reported their stress, sleeping behavior and daily activities using a smartphone application. A stress assessment algorithm was developed using only the physiological data collected with the wearable devices [10].

Other long-term studies aim to transfer the controlled laboratory assessment into real-world scenarios. Kyriakou et al. proposed a rule-based algorithm based on GSR and ST data. They combined empirical findings with expert knowledge to ensure transferability between laboratory settings and real-world field studies [42]. They were also able to detect urban stressors such as traffic congestion, dangerous driving situations, and crowded areas such as tourist attractions.

More recently, A micro patch was developed to monitor sweat on the fingertip along with heart rate and ambient temperature to determine stress events during the day (e.g., public speaking during a live-streamed academic conference or while teaching a class). While the biomarker signals look promising, a more thorough evaluation of this patch in combination with stress detection algorithm was left out of scope [43].

In long-term, real-world studies, sensor data is accompanied by a smartphone survey for collecting information about possible stress events. The answers in these surveys contain information regarding the participant’s mood or sleeping behavior. In some works, the stress recognition models use these additional features as one blob of additional context data. In others, they have left aside this information due to the low quality of the surveys. To our knowledge, no published research has reported the influence of specific context information on the models’ performance. Context information is however necessary to further improve the detection models and provide new insights for the treatment of stress-related diseases.

### Stress detection using context information

There are very few studies that consider context information for the task of stress detection. Many obtain this information either by means of questionnaires or using preliminary forms to analyze the integrity profile of the person who experiences stress [44]. As mentioned in the introduction questionnaires or diary based contextual information have the additional drawback of being inaccurate or biased as they can be subjectively interpreted [14].

Other studies investigated additional activities and additional observational information such as weather or sunlight as contextual information [45, 46]. These methodologies were evaluated on a very low number of participants and most of the time, the contextual data was provided in one big blob to the learning models without analyzing which context features lead to improved stress detection scores.

Even though it is clear that there is more to detecting stress than just momentarily physiological biosignals, there are very few studies considering the context in which these biosignals present themselves. This is the case, partially due to the difficulty of obtaining this information, as it requires and relies on a lot of human effort, which has been shown to be posing burden to people and is moreover recall and/or confirmation biased.

## Dataset

**Fig. 1:**
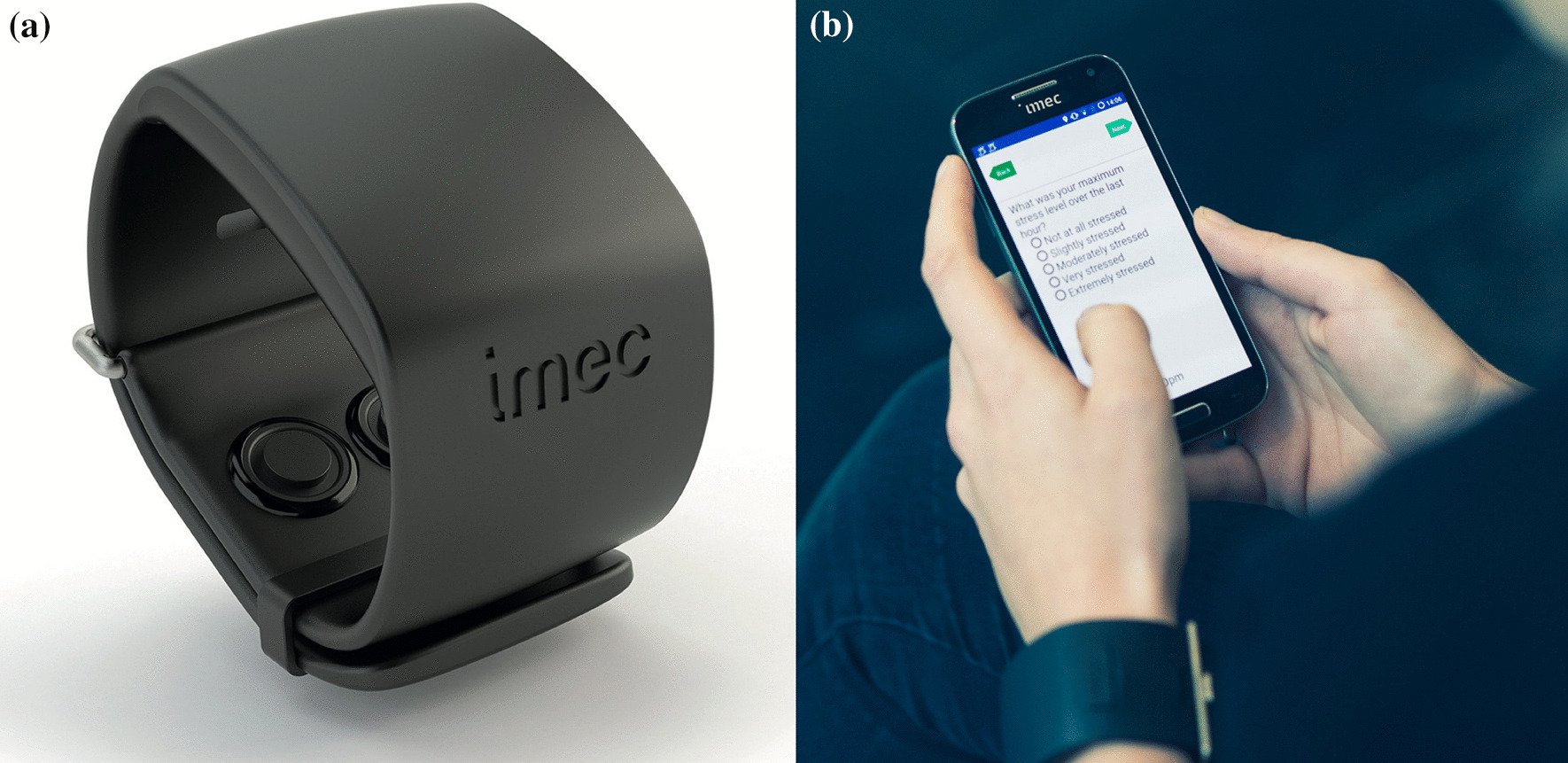
Left **a** the imec chillband wearable, right **b** the annotation app built to annotate hourly based stress levels, but also other contextual information

For this research, the dataset of [10] is used so the added value of the available context information can be addressed. Imec’s Stress in the Work Environment (SWEET) study captured data from more than 1002 people. It is the first large-scale study that used wearables to establish a link between mental stress and physiological symptoms in daily life. All participants wore the wearable device for 5 days starting from Thursday to capture data during weekends and the Monday rush. The wearable used in this study is the imec’s chillband, as shown in Fig. 1a. This device contains three different sensors: a 3-axis accelerometer sensor capturing the wrist’s motion at 32 Hz, a temperature sensor measuring the skin temperature at 4 Hz and a Galvanic Skin Conductance (GSR) sensor measuring changes in sweat gland activity at 4 Hz. As reported in [10], this device is able to provide 96.3 ± 2.2% good quality physiological data (good measured quality index in $\ge$80% of data points in a 5 min window).

The participants used a smartphone application, as shown in Fig. 1b to report their hourly stress levels and additional context information such as activity, food intake and sleeping behavior. In this section, we give more details on both the raw features derived from the wearable as well as more information on the self-reported data. An overview of all the data is provided in Table 1.

**Table 1:** Overview of all the features derived from the Chillband wearable and mobile app

Input	Data	Features per hour
Wearable	Accelormeter	73 tsfresh features (for each axis)
	GSR	73 tsfresh features
	Skin Temp.	73 tsfresh features
Mobile APP	Activity	Boolean multilabel: sitting, standing, walking, lying_down, running
	Sleep	Two timestamps: Time To Bed, Get Up and a derived time interval (duration)

### Physiological features

Five different time series represent the raw data captured by the wearable, one for each accelerometer axis, one for the skin temperature and one for the GSR values. Since the SWEET participants reported stress levels every hour, features were extracted using a window of 1 h. While previous evaluations on this SWEET study [10] used manually extracted features, we took advantage of recent advances in time series feature extraction tools such as the tsfresh Python package [47] to extract a large number of time series characteristics from each hour of the data. In total, the default tsfresh package extracted 73 different features for each signal. An overview of these features can be found in the Additional file 1: A.

### Self-reported data

In this study, the participants labeled their stress level every hour using self-reporting. Five different classes, from no stress to severe stress, were annotated. Similar to the previous stress study [10], we limited this five-level scale to three categories (by combining the highest three stress levels). Additionally, the participants were required to select their main activities every hour. Five different activities were listed: lying down, sitting, standing, walking, running, and biking. The participants could choose none, one or more of these activities per hour. Every morning, the app asked the participants to register their sleeping behavior, i.e., the time they went to bed and the time they woke up in the morning. Based on these two times, the duration of a participant’s sleep could be derived. Additionally, the app also asked to indicate a subjective quality of sleep score.

Beside sleep and activity annotations, the participants could also indicate their food intake and also additional remarks could be reported. The more subjective features and food intake were neglected in this study, as they had no to limited influence on any of the physiological measured wearable sensor values.

## Automated context retrieval

Even though a useful mobile app was available to select and annotate a lot of contextual information, annotating all this information is a rather tedious and cumbersome task. As a result, not all participants delivered a high-quality dataset. Moreover, the participants were unable to correctly or in detail remember the context leading to incorrect annotations, e.g. incorrect annotations of the time they went to sleep or annotate awake moments and toilet visits during the night. To reduce this subjective labeling approach not matching the objective observations, we designed two ML models to capture human activities and sleeping behavior automatically.

### Human Activity Recognition (HAR)

Providing accurate and suitable information on people’s activities and behaviors is one of the essential tasks in pervasive computing [48]. Innumerable applications use HAR directly or as domain context enrichment in medicine, security and entertainment.

There exist many different HAR models and techniques to detect a wide variety of activities [49]. Our proposed solution is designed on the same Chillband wearable used within the SWEET study and detects three different human activities: sedentary (sitting and standing), walking and biking. Sitting, standing, walking and biking, combined with sleeping were the top 5 most registered activities within the self-reported SWEET questionnaires. The goal of our HAR model is to keep the human labeling bias to a minimum while automatically detecting these activities. This section gives an overview of the dataset on which we have trained our HAR model, the ML pipeline and the achieved results.

#### HAR dataset

As the SWEET dataset only contains self-labeled activities per hour, another dataset was used to train the HAR. Our HAR model used a benchmark dataset consisting of data collected from 37 different participants in an uncontrolled environment [50]. Each subject wore a ChillBand for about 8 h and was free to do what they normally do. They also had a GoPro camera attached to their chest so the video data could be used to determine the exact timing and label of each performed activity.

A broad range of activities was annotated in this dataset: sitting, dynamic sitting, standing, dynamic standing, walking upstairs, walking downstairs, walking, running and cycling. We grouped all sitting, dynamic sitting, standing and dynamic standing activities in the sedentary class. The walking upstairs, walking downstairs, walking and running activities were all grouped together and considered as walking in general.

The ChillBand data captured for each person during these 8 h was stored offline for evaluation purposes and contained GSR, 3-axis accelerometer and skin temperature data. Activities with a duration less than 1 min were removed from this dataset. For the remaining dataset, we segmented all five signals into sliding windows of 15 s and with 50% overlap as the labels were originally also defined for every 15 s and a 50% gap was recommended [50]. In total, the HAR model was trained on 90,243 samples, of which 85,299 are sedentary, 2961 walking and 1983 cycling.

#### HAR methodology

Before extracting informative features, we pre-processed the accelerometer and GSR signal. To each of the accelerometer axis and their euclidean norm, we applied a butterworth bandpass filter of 4th order, with 0.3 and 15 as the low and high cutoff frequencies respectively. For each 15 s time window, we extracted a total of 228 statistical features, for the accelerometer features in both time and frequency domain, while for both the GSR and skin temperature signals only in the time domain (features are available in Additional file 1: B). Extraction of appropriate features was mainly based on literature study that describe features that are important within the activity detection domain. Besides time domain features for all sensors, we also consider frequency domain features, such as dominant frequency, from the accelerometer signal as they have been shown to discriminate activities well [51]. Frequency domain features from the GSR and ST signals make less sense as there are no repetitive patterns in these signals when considering windows of 15s. Before extracting the features, we z-normalized the GSR signal by subtracting the mean and dividing by the standard deviation. We then split the normalized signal into its phasic and tonic components [52]. For each component the corresponding features were calculated.

After preprocessing and feature engineering, the dataset was split into a train set (31 disjoint subjects) and a test set (6 disjoint subjects). We trained a Catboost (gradient boosting on decision trees) model for this 3-class classification problem and adopted a group 5 fold strategy, in which each fold has 25 (or 24) disjoint train subjects, and 6 (or 7) disjoint validation subjects, to tune the hyper-parameters on the training set. Given that our dataset is highly imbalanced (as the sedentary class is the majority class, being almost 95% of the whole dataset, while walking is about 3% and cycling 2%), we had to use an appropriate strategy to mitigate this problem. Since the class distribution is similar across all users and using a strictly stratified-group-5-fold CV could lead to imbalanced folds, we decided to use a standard group-5-fold. To address the data imbalance we used class-weights when training the model. The final model predicts the activity for every window of 15 s. Table 2 shows for each fold the amount of train and validation subjects as well as the number of samples per class. In the last row the number of subjects and the number of samples per class in the train and hold-out test set for the final model are shown. We can see that in each fold there are samples from each activity in both the training and validating split.

**Table 2:** Number of subjects and samples per class, for train/validation in each fold and train/test for final model

	Train				Test			
	Subjects	Sedentary	Walking	Cycling	Subjects	Sedentary	Walking	Cycling
Fold1	25	58,224	1849	1454	6	14,313	708	409
Fold2	25	57,848	2101	1445	6	14,690	456	418
Fold3	24	58,585	2150	1474	7	13,952	407	389
Fold4	25	57,770	2149	1484	6	14,767	408	379
Fold5	25	57,722	1979	1595	6	14,815	578	268
Final	31	72,537	2557	1863	6	12,762	404	120

#### HAR results

**Fig. 2:**
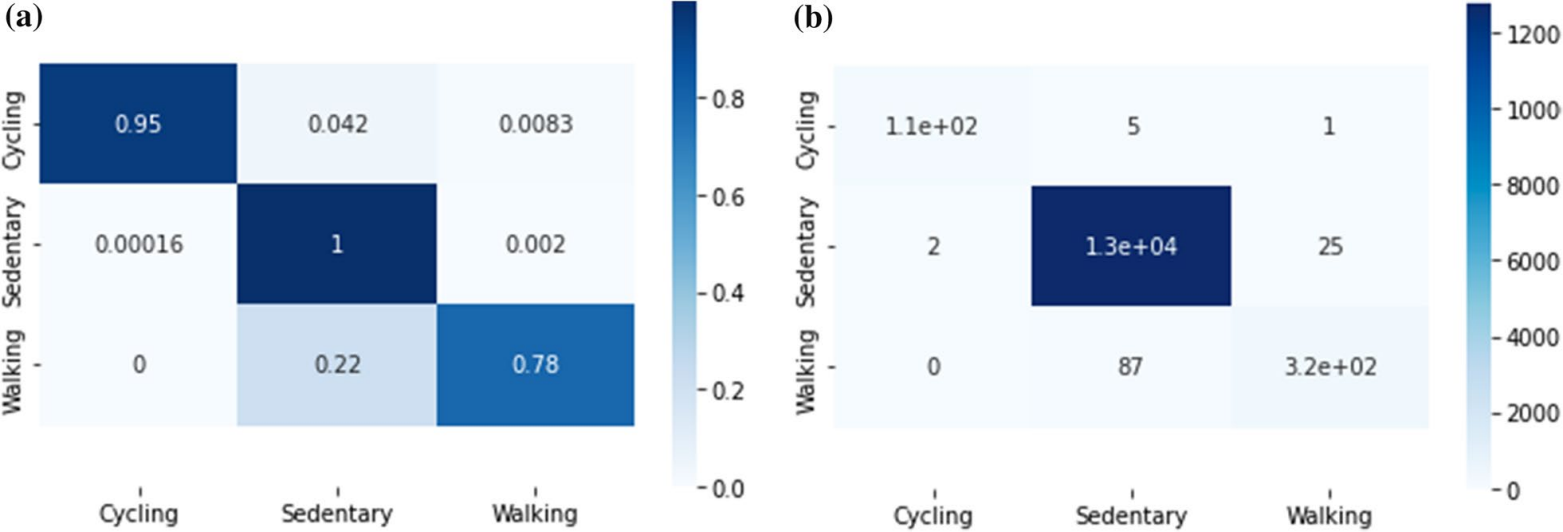
Left **a** normalized and right **b** absolute-numbers confusion matrix from the predictions on the hold-out test set

On the hold-out test split, we achieved results as shown in Table 3 and Fig. 2:

**Table 3:** HAR Catboost results on the hold out test set

	Precision	Recall	F1-score	Support
Sedentary	0.99	1	1	12,762
Walking	0.92	0.78	0.85	404
Cycling	0.98	0.95	0.97	120

The results show that for all three classes, we achieve high precision. For the walking class, the recall is lower, which results in an F1 score below 0.9. This can be clarified by the fact that we aggregated the rather diverse dynamic walking activities. The results give enough evidence that our model can be used to predict activities and use these as additional context within the $\mathsf {SWEET}$ study, and compare with the results when users self-label their activities. This was also the goal of this paper, rather than building the best performing HAR model.

### Sleep detection

As shown in Fig. 1b, the SWEET study participants were asked to indicate their sleeping behavior, i.e., start, stop and quality of their sleep. Instead of annotating these values manually through the app, it would be more beneficial if they can be automatically derived from the wearable’s sensor values. Also here, the SWEET dataset did not contain labeled sleep data. But instead of building a new dataset and training a supervised ML model (as we did for the HAR in "Human Activity Recognition (HAR)" section), we designed an unsupervised sleep detection model directly on the SWEET dataset.

#### Sleep detection methodology

**Fig. 3:**
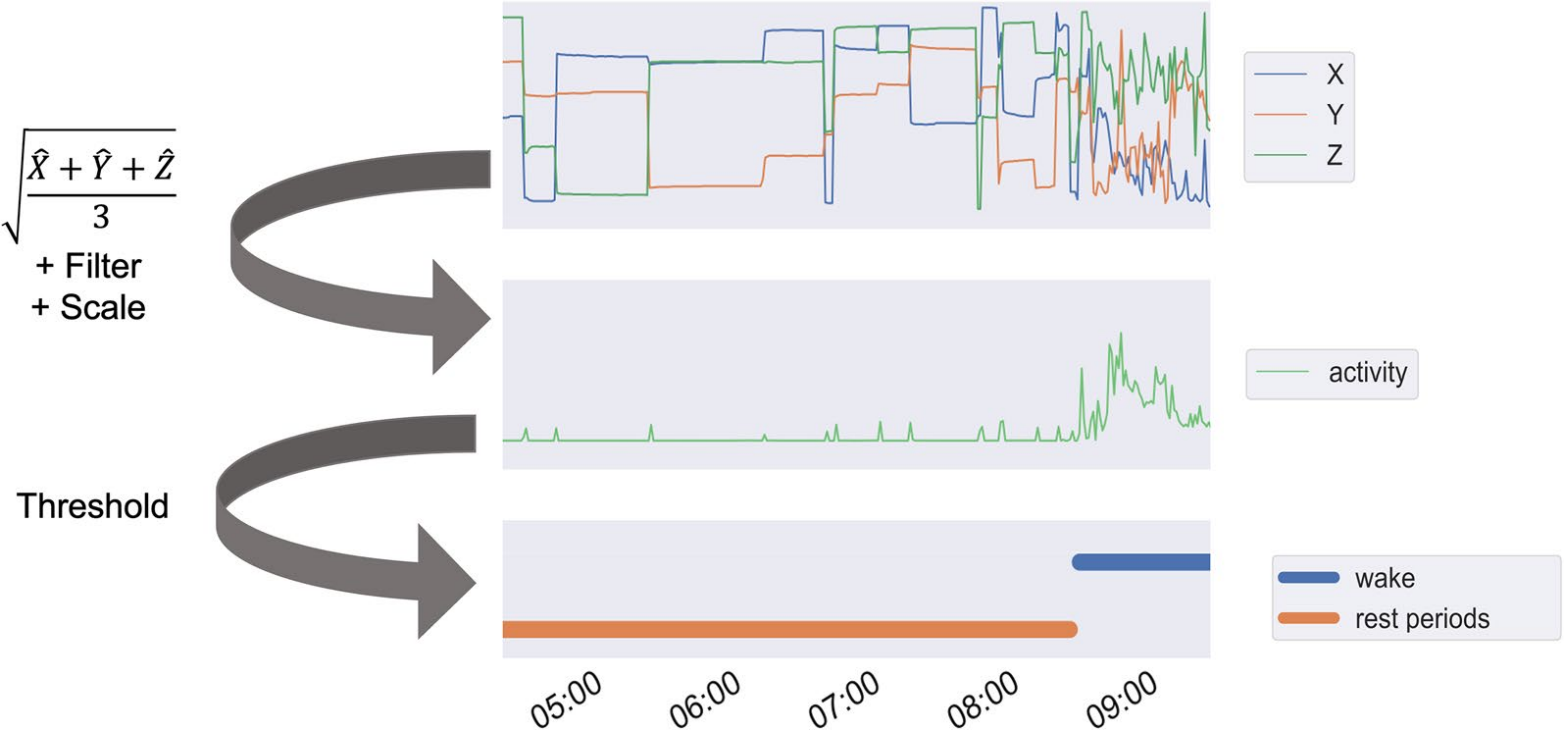
Overview of the performed steps to determine the wake and rest periods based on a three-axis accelerator sensor

The unsupervised sleep detection model uses an activity index [53], which is calculated from the raw accelerometer signal. This activity index is defined as the square root of the mean variance over a rolling window of 10 min: we calculate the variance for each axis along the time dimension, and then we take the mean across the three axes. This yields one value for a window of 10 min. This value is later on filtered using a butterworth bandpass filter of third order and scaled to a range from 0 to 1. The more active a user was at a given moment in time, the higher the activity index. While sleeping, this activity index will be lower compared to periods in which the participant is awake. To define the threshold, a sleep and wake state detection methodology was designed based on a heuristic model around the automatic scoring algorithm of Cole et al. [54]. The algorithm defines a score based on the activity index, indicating how certain these values are associated with an awake or sleep period. Combining all these scores reveals the sleep pattern. Figure 3 shows these steps on a part of a signal. To determine the begin and end period of the sleep patterns, the binary segmentation algorithm of the Python package ruptures was executed on the activity index signal [55]. This segmentation algorithm searches for 2 change points within the activity index signal, with a defined minimal amount of samples between these two change points spawning a range of 5 h.

#### Sleep detection results

Since our sleep detection uses heuristics and unsupervised techniques, we evaluated it directly on the SWEET dataset. Sleeping with the wearable was not mandatory during the SWEET study and only 662 unique users did so. We used 1777 nights in total during our evaluations. The results of this unsupervised approach compared to the self-labeled sleep annotations are discussed in the SWEET experiments below.

## Experiments and results

For the experimental evaluation, we defined the stress detection task as a 3 class classification problem (no, medium and high stress), similarly as was done in the SWEET study and also used the data from this study. We trained four different models:

1. A model trained on only the physiological tsfresh features as explained in "Physiological features" section
2. A model trained on the physiological tsfresh features and activity features as context information.
3. A model trained on the physiological tsfresh features and sleep features as context information.
4. A model trained on the physiological tsfresh features and both the activity and sleep features.

The activity features consist of three values, each corresponding to the number of minutes spent on the corresponding activity in the given hour. There are also three sleep features: start hour of sleep, end hour of sleep and duration of sleep.

Two different experiments evaluate the added value of using this context information in stress detection models. In the first experiment, we extracted the context features only from the self-reported activity and sleep information within the SWEET study. In the second experiment, we automatically derived the activity and sleep information, as described in "Automated context retrieval" section, on the sensor data collected within the SWEET study. We then extracted features from this information and used them in our models. Finally, we compared both results to evaluate the impact of automatically monitoring versus self-reported context.

To test whether context information influences the prediction accuracy, we consciously chose to use the same algorithm for all the evaluations, more specifically CatBoost, as (1) Gradient Boosted trees, such as CatBoost, are considered, in general, to outperform Random Forests, (2) CatBoost yields a robust model without having to tune too many hyperparameters, and (3) CatBoost is fast in both training and inference.

### Self-reported context

From the 1002 participants, 827 had provided complete context information that could be linked to reported stress events. To avoid the influence of missing values, the stress events were aligned with both the activity labels of the matching last hour and the sleep patterns of the previous night. Since the participants only indicated which activity/activities they mainly performed in the last hour, but not specified the duration, we split the 60 min in the hour equally among all the reported activities. For example, if the person indicated Sedentary and Cycling activity, then Sedentary and Cycling were each assigned a corresponding value of 30, while Walking was assigned a 0 value. The sleep features, time going to bed and time waking up, were literally taken from the reports, and the duration of the sleep was derived from these two times. These 827 users reported in total 4895 stress events (2556 labelled as no stress, 1674 as medium stress and 665 as high stress). These aligned stress events form the final dataset used for training the four models discussed above. We chose to use Catboost to combine both numerical and the self-reported or derive context labels as ML algorithm. To reduce the bias introduced by the participants, we performed GroupKfold cross-validation with 3 folds, 1 fold for training, 1 fold for validation and 1 for testing. The validation group was used for optimizing the tree depth and perform early stopping based on the Weighted Kappa metric. The same splits were kept over all four models to make a fair comparison. All models had a maximum iteration of 10,000, but we stopped the training process earlier when no improvement on the validation set could be noticed for 150 consecutive iterations. We provided class weights to address the class imbalance in our dataset.

We calculated four different evaluation metrics which we used to analyse the influence of the context features on the stress models’ performance:

- Accuracy metric: the number of correctly predicted stress labels out of all the stress labels;
- Cohen Kappa score: measures how closely the stress labels classified by our model resembles the ground truth, compared to the accuracy of a random classifier measured by the expected accuracy;
- Weighted F1 score: the harmonic mean of the precision and recall.
- Weighted F1 compared to dummy: difference between the calculated weighted F1 prediction to the weighted F1 calculated by a dummy classifier predicting the majority class.

The results are visualised in Table 4. We observe that incorporating the activity information into our baseline model improves stress level classification. Not only the values of the metrics increase, but also the standard deviations decrease. Adding sleep features also improves our baseline model but slightly less than when incorporating the activity information. Incorporating both the activity and sleep features yields the best predictive performance.

**Table 4:** Results comparing the influence of labelled context features on the assessment of stress levels

Model	Accuracy (std)	Cohen Kappa (std)	Weighted F1 (std)	Compared to dummy (std)
Baseline (physio)	40.57 (3.48)	7.80 (1.98)	42.11 (3.08)	6.31 (2.53)
Baseline + activity	43.09 (0.67)	9.75 (0.09)	44.47 (0.44)	8.68 (0.11)
Baseline + sleep	42.47 (0.72)	9.51 (0.12)	43.88 (0.59)	8.09 (0.03)
Baseline + activity + sleep	45.52 (2.58)	11.19 (1.58)	46.03 (2.12)	10.23 (2.68)

### ML-based context

To show the added value of using ML derived context, we followed a similar approach to the one discussed above. To derive the context information by using ML, we need sensor data. However, participants did not use the wearable device every time they provided context information (be it activity or sleep information). This limited our dataset to 380 participants, mainly because we had sensor data from only 1777 nights compared to more than 4000 sleep reports.

To derive the activity information, the sensor data was prepared in the same manner as explained in "HAR methodology" section, i.e., we pre-processed the data in 15s sliding windows, with 50% overlap, and calculated the necessary features for the activity recognition. After applying inference, we have the activity probabilities for every 7.5 s. We then aggregate these predictions per hour as follows: first we aggregate per minute, by adding the probabilities from each prediction and take the activity with the highest probability sum as the final prediction for that minute. We then count the 1-min predictions for each activity in the given hour and these become the activity features as described in "Experiments and results" section. Similarly, to derive the sleep features, we prepare and process the accelerometer data of a whole day, starting from 1 pm till 1 pm the next day, as explained in "Sleep detection methodology" section. We then apply the same algorithm (see "Sleep detection methodology" section) which yields the start and end time of the resting period. These two times are then used to calculate the duration of the sleep, resulting in three sleep features (start, end, duration).

In this experiment, the dataset consisted of 618 stress events (320 labelled as no stress, 217 as medium stress and 81 as high stress). Similarly to "Self-reported context" section, also here Catboost models were trained using the same hyper-parameters and parameter tuning. We also kept the same train, validation and test split to achieve a fair comparison.

The same four different metrics described in the previous section were also here calculated for this experiment. We additionally compare the results obtained by using ML derived context information with results obtained by using self-reported context information. The results are presented in Table 5.

**Table 5:** Results comparing the labelled contextual features with the derived ML ones

Model		Accuracy (std)	Cohen Kappa (std)	Weighted F1 (std)	Compared to dummy (std)
Physio (baseline)		40.24 (1.60)	2.05 (1.10)	40.58 (0.81)	5.26 (2.44)
Physio + activity	Labelled	38.79 (1.47)	− 0.94 (0.51)	39.22 (0.75)	3.90 (4.00)
	Predicted	43.40 (2.16)	1.14 (1.02)	42.19 (1.54)	6.86 (1.70)
Physio + sleep	Labelled	43.96 (1.59)	4.03 (1.25)	43.18 (0.41)	7.85 (2.83)
	Predicted	44.20 (2.97)	3.00 (2.89)	43.06 (2.42)	7.74 (0.83)
Physio + activity + sleep	Labelled	42.09 (5.08)	3.42 (4.02)	41.88 (4.17)	6.55 (0.92)
	Predicted	41.69 (4.35)	1.49 (0.78)	41.48 (2.93)	6.15 (0.31)

The baseline approach for this experiment only uses physiological data and is therefore entirely the same for both the labelled and predicted context-aware models. The baseline results are, however, less accurate than in Table 5 due to the reduction of available training data. By analysing the comparison to dummy results, we encounter that adding contextual labelled features or ML derived ones hold similar results except that the standard deviation is reduced significantly. Predicted activities improve the model significantly over self-labelled activities, but combining all predicted features does not reflect this advantage. This can be clarified by the implicit duplicate information within the sedentary activities and sleep duration. The sleeping behaviour also indirectly influences the stress responses, while the activities are more reactive.

## Discussion

The main goal of this research was to assess the added value of using context in stress detection based on wearable device data. Table 4 shows the benefits of using both simple activity features and sleep patterns in combination with physiological wearable features to determine the stress levels. The improved performance is mainly due to the reduction of false positives (predictions of stress levels when the labels indicated that there was no stress). Physiological features are similar when a person is experiencing stress and when they perform an intensive activity: there is an increase in both GSR and skin temperature values. By providing activity information, the model can learn to better derive stressful events from pure physical activities. Sleep deprivation is also a factor which influences stress [19]. Therefore taking into account the user’s sleeping behaviour also positively affects the performance of our models.

Providing contextual information is valuable, but it requires a lot of human effort. While filling out multiple questionnaires is common in experimental settings, they are not useful and too cumbersome for daily life assessments. Therefore, the amount of self-reported context should be limited when possible. As shown in Table 5, the number of manual annotations for both activities and sleep patterns can be limited to zero by using ML based activity recognition and sleep detection models.

Besides the reduction of human effort, using automatic context retrieval holds additional advantages:

- *Fine-grained detection* The activity labels within the SWEET study only indicated whether or not a certain activity was performed during the last hour. The activity recognition model also indicated the duration of these activities and could better define the active periods of a participant during the day.
- *Improved accuracy* Annotating the time one fell asleep and the moment one woke up is relatively hard and introduces some subjective bias. By detecting the sleeping patterns using wearable data, we avoid the subjectivity. On average, the detected sleep duration differed 48 min (std: 52 min) from the self-reported one with more faults for wake up (mean 34 min, with std: 41 min) than the time to bed times (mean 32 min and std: 35 min).
- *Information gain* In order to not overload the participants, most questionnaires in the SWEET study were asked between 7:00 and 22:00 during the SWEET study. Activity annotations before or after this time range are therefore missing. Not answering the questionnaire also leads to missing annotations. Participants sometimes failed to provide answers due to different reasons: being busy, lack of motivation, not receiving the notification on time, etc. Automatically retrieving this information reduces the need for these questionnaires and the accompanying missing values.

The main drawback of automatically retrieving context is the need for sensor data. However, as the participants already have to wear wearable for the stress estimation, the additional impact is limited. Compared to video and sound estimation techniques, the used wearable approach is also less invasive and more privacy-aware. The main reason why here only 618 stress events were available in Table 5 is because it was not required to sleep with the wearable in the SWEET study. Having shown the benefit of having the sleep context info, participants can choose to either label their sleep manually or wear the wearable overnight.

Since the goal of this paper was to research the influence of context on stress detection, rather than create and present a stress detection model by means of tuning all possible hyperparameters, we did not go in depth in certain steps such as feature selection and testing different algorithms. Instead, we chose a fixed set of physiological features and a single ML algorithm to assure fair comparison on the influence of context on stress detection in all our experiments.

For all of the ML models we used Gradient Boosted Trees (GBT), more specifically CatBoost, which have fast training time (order of few minutes) and inference time (order of just few seconds). This makes GBTs adequate algorithms for real world application and edge computing: their fast inference time allows for near immediate assessment to the user, who can be then timely warned of their stress onset, allowing for appropriate action. GBTs can moreover be explainable, in contrast to more complex models such as Artificial Neural Networks, which is highly desired in the field of medical decision making.

## Conclusion

To assess the added value of contextual information during the detection of stress, we set up experiments in which we combined physiological wearable data and contextual features. These contextual features are derived from hourly activity and daily sleeping information. Both activity features and sleeping patterns improve the stress detection model by either reducing the number of false positives or by providing additional information to predict the correct stress level.

This study also examined how this contextual information can be derived automatically using ML. Wearable data was used to derive activities and detect sleeping patterns. Incorporating ML-derived context information, especially the performed activities, leads to higher stress detection accuracy, as the label quality of the detected activities is higher and more fine-grained than when using the self-reported label which is on a 1-h basis. Besides the impact on predictive accuracy, the biggest gain is in moving away from human (subjective) labeling towards automated (objective) labels, which in turn lowers the effort burden on the participants, reducing chances for dropping out of studies and/or monitoring.

Future work can resolve these problems by encouraging people to use the wearable as much as possible and derive even more information using context retrieval models. More sleep information, such as awakenings, or activity intensity, can be incorporated when the wearable is worn when asleep. The research evaluating the benefit of contextual information on the prediction of stress can also be used in other domains, such as for mood detection or the detection of headache events.

## Supplementary Information

**Additional file 1: A** - Tsfresh features and **B** - Wearable statistical features.

## Data Availability

The data from the SWEET study [10] are available on request to the corresponding author of that study. The data are not publicly available due to them containing information that could compromise research subject privacy.
