# Supplementary material for: Assessing the added value of context during stress detection from wearable data
Source: BMC Med Inform Decis Mak. 2022 Oct 15;22:268. doi: 10.1186/s12911-022-02010-5 (PMC9571684; doi:10.1186/s12911-022-02010-5)
Supplement: Supplementary file 1 — Additional file 1: A - Tsfresh features and B - Wearable statistical features. [file 12911_2022_2010_MOESM1_ESM.pdf]

Additional file

A: tsfresh features:

abs_energy(x)	Returns the absolute energy of the time series which is the sum over the squared values
absolute_sum_of_changes(x)	Returns the sum over the absolute value of consecutive changes in the series x
agg_autocorrelation(x, param)	Calculates the value of an aggregation function f_agg
agg_linear_trend(x, param)	Calculates a linear least-squares regression for values of the time series that were aggregated over chunks versus the sequence from 0 up to the number of chunks minus one.
approximate_entropy(x, m, r)	Implements a vectorized Approximate entropy algorithm.
ar_coefficient(x, param)	This feature calculator fits the unconditional maximum likelihood of an autoregressive AR(k) process.
augmented_dickey_fuller(x, param)	The Augmented Dickey-Fuller test is a hypothesis test which checks whether a unit root is present in a time series sample.
autocorrelation(x, lag)	Calculates the autocorrelation of the specified lag, according to the formula [1]
benford_correlation(x)	Useful for anomaly detection applications [1][2].
binned_entropy(x, max_bins)	First bins the values of x into max_bins equidistant bins.
c3(x, lag)	This function calculates the value of $\frac{1}{n-2lag}\sum_{i=1}^{n-2lag} x_{i+2\cdot lag}\cdot x_{i+lag}\cdot x_i$
change_quantiles(x, ql, qh, isabs, f_agg)	First fixes a corridor given by the quantiles ql and qh of the distribution of x.
cid_ce(x, normalize)	This function calculator is an estimate for a time series complexity [1] (A more complex time series has more peaks, valleys etc.).
count_above(x, t)	Returns the percentage of values in x that are higher than t
count_above_mean(x)	Returns the number of values in x that are higher than the mean of x

count_below(x, t)	Returns the percentage of values in x that are lower than t
count_below_mean(x)	Returns the number of values in x that are lower than the mean of x
cwt_coefficients(x, param)	Calculates a Continuous wavelet transform for the Ricker wavelet, also known as the "Mexican hat wavelet" which is defined by $\frac{2}{\sqrt{3a}\pi^{\frac{1}{4}}}\left(1-\frac{x^2}{a^2}\right)\exp\left(-\frac{x^2}{2a^2}\right)$
energy_ratio_by _chunks(x, param)	Calculates the sum of squares of chunk i out of N chunks expressed as a ratio with the sum of squares over the whole series.
fft_aggregated(x, param)	Returns the spectral centroid (mean), variance, skew, and kurtosis of the absolute fourier transform spectrum.
fft_coefficient(x, param)	Calculates the fourier coefficients of the one-dimensional discrete Fourier Transform for real input by fast
first_location_of_maximum(x)	Returns the first location of the maximum value of x.
first_location_of_minimum(x)	Returns the first location of the minimal value of x.
fourier_entropy(x, bins)	Calculate the binned entropy of the power spectral density of the time series (using the welch method).
friedrich_coefficients(x, param)	Coefficients of polynomial h(x), which has been fitted to
has_duplicate(x)	Checks if any value in x occurs more than once
has_duplicate_max(x)	Checks if the maximum value of x is observed more than once
has_duplicate_min(x)	Checks if the minimal value of x is observed more than once
index_mass_quantile(x, param)	Those apply features calculate the relative index i where q% of the mass of the time series x lie left of i.
kurtosis(x)	Returns the kurtosis of x (calculated with the adjusted Fisher-Pearson standardized moment coefficient G2).
large_standard_deviation(x, r)	Boolean variable denoting if the standard dev of x is higher than 'r' times the range = difference between max and min of x.

last_location_of_maximum(x)	Returns the relative last location of the maximum value of x.
last_location_of_minimum(x)	Returns the last location of the minimal value of x.
lempel_ziv_complexity(x, bins)	Calculate a complexity estimate based on the Lempel-Ziv compression algorithm.
length(x)	Returns the length of x
linear_trend(x, param)	Calculate a linear least-squares regression for the values of the time series versus the sequence from 0 to length of the time series minus one.
linear_trend_timewise(x, param)	Calculate a linear least-squares regression for the values of the time series versus the sequence from 0 to length of the time series minus one.
longest_strike_above_mean(x)	Returns the length of the longest consecutive subsequence in x that is bigger than the mean of x
longest_strike_below_mean(x)	Returns the length of the longest consecutive subsequence in x that is smaller than the mean of x
max_langevin_fixed_point(x, r, m)	Largest fixed point of dynamics :math:argmax_x h(x)=0‘ estimated from polynomial h(x),
maximum(x)	Calculates the highest value of the time series x.
mean(x)	Returns the mean of x
mean_abs_change(x)	Returns the mean over the absolute differences between subsequent time series values which is
mean_change(x)	Returns the mean over the differences between subsequent time series values which is
mean_second_derivative_central(x)	Returns the mean value of a central approximation of the second derivative
median(x)	Returns the median of x
minimum(x)	Calculates the lowest value of the time series x.
number_crossing_m(x, m)	Calculates the number of crossings of x on m.
number_cwt_peaks(x, n)	This feature calculator searches for different peaks in x.
number_peaks(x, n)	Calculates the number of peaks of at least support n in the time series x.

partial_autocorrelation(x, param)	Calculates the value of the partial autocorrelation function at the given lag.
percentage_of_reoccurring_datapoints_to_all_datapoints(x)	Returns the percentage of unique values, that are present in the time series more than once.
percentage_of_reoccurring_values_to_all_values(x)	Returns the ratio of unique values, that are present in the time series more than once.
permutation_entropy(x, tau, dimension)	Calculate the permutation entropy.
quantile(x, q)	Calculates the q quantile of x.
range_count(x, min, max)	Count observed values within the interval [min, max).
ratio_beyond_r_sigma(x, r)	Ratio of values that are more than r*std(x) (so r sigma) away from the mean of x.
ratio_value_number_to_time_series_length(x)	Returns a factor which is 1 if all values in the time series occur only once, and below one if this is not the case.
sample_entropy(x)	Calculate and return sample entropy of x.
set_property(key, value)	This method returns a decorator that sets the property key of the function to value
skewness(x)	Returns the sample skewness of x (calculated with the adjusted Fisher-Pearson standardized moment coefficient G1).
spkt_welch_density(x, param)	This feature calculator estimates the cross power spectral density of the time series x at different frequencies.
standard_deviation(x)	Returns the standard deviation of x
sum_of_reoccurring_data_points(x)	Returns the sum of all data points, that are present in the time series more than once.
sum_of_reoccurring_values(x)	Returns the sum of all values, that are present in the time series more than once.
sum_values(x)	Calculates the sum over the time series values
symmetry_looking(x, param)	Boolean variable denoting if the distribution of x looks symmetric.
time_reversal_asymmetry_statistic(x, lag)	This function calculates the value of
value_count(x, value)	Count occurrences of value in time series x.

variance(x)	Returns the variance of x
variance_larger_than_standard_deviation(x)	Boolean variable denoting if the variance of x is greater than its standard deviation.
variation_coefficient(x)	Returns the variation coefficient (standard error / mean, give relative value of variation around mean) of x.

B: Wearable statistical features:

GSR_SMNA_0	Sparse SMNA driver of first phasic component
GSR_SMNA_1	Sparse SMNA driver of second phasic component
GSR_SMNA_2	Sparse SMNA driver of third phasic component
GSR_SMNA_3	Sparse SMNA driver of fourth phasic component
GSR_SMNA_4	Sparse SMNA driver of fifth phasic component
GSR_SMNA_5	Sparse SMNA driver of sixth phasic component
GSR_SMNA_6	Sparse SMNA driver of seventh phasic component
GSR_SMNA_7	Sparse SMNA driver of eight phasic component
GSR_SMNA_8	Sparse SMNA driver of ninth phasic component
GSR_SMNA_9	Sparse SMNA driver of tenth phasic component
GSR_SMNA_10	Sparse SMNA driver of eleventh phasic component
GSR_SMNA_11	Sparse SMNA driver of twelfth phasic component
GSR_SMNA_12	Sparse SMNA driver of thirteenth phasic component
GSR_SMNA_13	Sparse SMNA driver of fourteenth phasic component
GSR_SMNA_14	Sparse SMNA driver of fifteenth phasic component
GSR_linear_drift_offset	Offset of linear drift term
GSR_linear_drift_slope	Slope of linear drift term
GSR_mean	Mean of EDA value
GSR_tonic_auc	AUC of tonic component
ACC_x_mean	Mean of x-axis accelerometer values
ACC_y_mean	Mean of y-axis accelerometer values
ACC_z_mean	Mean of z-axis accelerometer values
ACC_x_median	Median of x-axis accelerometer values
ACC_y_median	Median of y-axis accelerometer values
ACC_z_median	Median of z-axis accelerometer values
ACC_x_min	Minimum of x-axis accelerometer values

ACC_y_min	Minimum of y-axis accelerometer values
ACC_z_min	Minimum of z-axis accelerometer values
ACC_x_max	Maximum of x-axis accelerometer values
ACC_y_max	Maximum of y-axis accelerometer values
ACC_z_max	Maximum of z-axis accelerometer values
ACC_x_std	Standard deviation of x-axis accelerometer values
ACC_y_std	Standard deviation of y-axis accelerometer values
ACC_z_std	Standard deviation of z-axis accelerometer values
ACC_x_var	Variance of x-axis accelerometer values
ACC_y_var	Variance of y-axis accelerometer values
ACC_z_var	Variance of z-axis accelerometer values
ACC_x_ptp	Range between maximum and minimum x-axis accelerometer values
ACC_y_ptp	Range between maximum and minimum y-axis accelerometer values
ACC_z_ptp	Range between maximum and minimum z-axis accelerometer values
ACC_x_absmean	Absolute mean of x-axis accelerometer values
ACC_y_absmean	Absolute mean of y-axis accelerometer values
ACC_z_absmean	Absolute mean of z-axis accelerometer values
ACC_x_skew	Skew of x-axis accelerometer values
ACC_y_skew	Skew of y-axis accelerometer values
ACC_z_skew	Skew of z-axis accelerometer values
ACC_x_kurt	Kurtosis of x-axis accelerometer values
ACC_y_kurt	Kurtosis of y-axis accelerometer values
ACC_z_kurt	Kurtosis of z-axis accelerometer values
ACC_x_iqr	Inter quartile range of x-axis accelerometer values
ACC_y_iqr	Inter quartile range of y-axis accelerometer values
ACC_z_iqr	Inter quartile range of z-axis accelerometer values
ACC_x_absarea	Absolute value of the area of x-axis accelerometer values

ACC_y_absarea	Absolute value of the area of y-axis accelerometer values
ACC_z_absarea	Absolute value of the area of z-axis accelerometer values
ACC_x_area	Area of x-axis accelerometer values
ACC_y_area	Area of y-axis accelerometer values
ACC_z_area	Area of z-axis accelerometer values
ACC_x_rms	Root mean square of x-axis accelerometer values
ACC_y_rms	Root mean square of y-axis accelerometer values
ACC_z_rms	Root mean square of z-axis accelerometer values
ACC_x_mean_crossings	Mean crossings of x-axis accelerometer values
ACC_y_mean_crossings	Mean crossings of y-axis accelerometer values
ACC_z_mean_crossings	Mean crossings of z-axis accelerometer values
ACC_x_dom_freq	Dominant frequency of x-axis accelerometer values
ACC_x_total_power	Sum of the total power of x-axis accelerometer values
ACC_x_power_skew	Skew of the power of x-axis accelerometer values
ACC_x_power_kurt	Kurtosis of the power of x-axis accelerometer values
ACC_x_power_mean	Mean of the power of x-axis accelerometer values
ACC_x_psd_0	First power spectral density component of x-axis accelerometer values
ACC_x_psd_1	Second power spectral density component of x-axis accelerometer values
ACC_x_psd_2	Third power spectral density component of x-axis accelerometer values
ACC_x_psd_3	Fourth power spectral density component of x-axis accelerometer values
ACC_x_psd_4	Fifth power spectral density component of x-axis accelerometer values
ACC_x_psd_5	Sixth power spectral density component of x-axis accelerometer values
ACC_x_psd_6	Seventh power spectral density component of x-axis accelerometer values
ACC_x_psd_7	Eight power spectral density component of x-axis accelerometer values

ACC_x_psd_8	Ninth power spectral density component of x-axis accelerometer values
ACC_x_psd_9	Tenth power spectral density component of x-axis accelerometer values
ACC_x_psd_10	Eleventh power spectral density component of x-axis accelerometer values
ACC_x_psd_11	Twelfth power spectral density component of x-axis accelerometer values
ACC_x_psd_12	Thirteenth power spectral density component of x-axis accelerometer values
ACC_x_psd_13	Fourteenth power spectral density component of x-axis accelerometer values
ACC_x_psd_14	Fifteenth power spectral density component of x-axis accelerometer values
ACC_x_psd_15	Sixteenth power spectral density component of x-axis accelerometer values
ACC_x_psd_16	Seventeenth power spectral density component of x-axis accelerometer values
ACC_y_dom_freq	Dominant frequency of y-axis accelerometer values
ACC_y_total_power	Sum of the total power of y-axis accelerometer values
ACC_y_power_skew	Skew of the power of y-axis accelerometer values
ACC_y_power_kurt	Kurtosis of the power of y-axis accelerometer values
ACC_y_power_mean	Mean of the power of y-axis accelerometer values
ACC_y_psd_0	First power spectral density component of y-axis accelerometer values
ACC_y_psd_1	Second power spectral density component of y-axis accelerometer values
ACC_y_psd_2	Third power spectral density component of y-axis accelerometer values
ACC_y_psd_3	Fourth power spectral density component of y-axis accelerometer values
ACC_y_psd_4	Fifth power spectral density component of y-axis accelerometer values
ACC_y_psd_5	Sixth power spectral density component of y-axis accelerometer values
ACC_y_psd_6	Seventh power spectral density component of y-axis accelerometer values
ACC_y_psd_7	Eight power spectral density component of y-axis accelerometer values

ACC_y_psd_8	Ninth power spectral density component of y-axis accelerometer values
ACC_y_psd_9	Tenth power spectral density component of y-axis accelerometer values
ACC_y_psd_10	Eleventh power spectral density component of y-axis accelerometer values
ACC_y_psd_11	Twelfth power spectral density component of y-axis accelerometer values
ACC_y_psd_12	Thirteenth power spectral density component of y-axis accelerometer values
ACC_y_psd_13	Fourteenth power spectral density component of y-axis accelerometer values
ACC_y_psd_14	Fifteenth power spectral density component of y-axis accelerometer values
ACC_y_psd_15	Sixteenth power spectral density component of y-axis accelerometer values
ACC_y_psd_16	Seventeenth power spectral density component of y-axis accelerometer values
ACC_z_dom_freq	Dominant frequency of z-axis accelerometer values
ACC_z_total_power	Sum of the total power of z-axis accelerometer values
ACC_z_power_skew	Skew of the power of z-axis accelerometer values
ACC_z_power_kurt	Kurtosis of the power of z-axis accelerometer values
ACC_z_power_mean	Mean of the power of z-axis accelerometer values
ACC_z_psd_0	First power spectral density component of z-axis accelerometer values
ACC_z_psd_1	Second power spectral density component of z-axis accelerometer values
ACC_z_psd_2	Third power spectral density component of z-axis accelerometer values
ACC_z_psd_3	Fourth power spectral density component of z-axis accelerometer values
ACC_z_psd_4	Fifth power spectral density component of z-axis accelerometer values
ACC_z_psd_5	Sixth power spectral density component of z-axis accelerometer values
ACC_z_psd_6	Seventh power spectral density component of z-axis accelerometer values
ACC_z_psd_7	Eight power spectral density component of z-axis accelerometer values

ACC_z_psd_8	Ninth power spectral density component of z-axis accelerometer values
ACC_z_psd_9	Tenth power spectral density component of z-axis accelerometer values
ACC_z_psd_10	Eleventh power spectral density component of z-axis accelerometer values
ACC_z_psd_11	Twelfth power spectral density component of z-axis accelerometer values
ACC_z_psd_12	Thirteenth power spectral density component of z-axis accelerometer values
ACC_z_psd_13	Fourteenth power spectral density component of z-axis accelerometer values
ACC_z_psd_14	Fifteenth power spectral density component of z-axis accelerometer values
ACC_z_psd_15	Sixteenth power spectral density component of z-axis accelerometer values
ACC_z_psd_16	Seventeenth power spectral density component of z-axis accelerometer values
ACC_smv_mean	Mean of combined axis accelerometer values
ACC_smv_low_mean	Mean of combined axis accelerometer low frequencies (0.3 to 2.5) values
ACC_smv_median	Median of combined axis accelerometer values
ACC_smv_low_median	Median of combined axis accelerometer low frequencies (0.3 to 2.5) values
ACC_smv_min	Minimum of combined axis accelerometer values
ACC_smv_low_min	Minimum of combined axis accelerometer low frequencies (0.3 to 2.5) values
ACC_smv_max	Maximum of combined axis accelerometer values
ACC_smv_low_max	Maximum of combined axis accelerometer low frequencies (0.3 to 2.5) values
ACC_smv_std	Standard deviation of combined axis accelerometer values
ACC_smv_low_std	Standard deviation of combined axis accelerometer low frequencies (0.3 to 2.5) values
ACC_smv_var	Variance of combined axis accelerometer values
ACC_smv_low_var	Variance of combined axis accelerometer low frequencies (0.3 to 2.5) values

ACC_smv_ptp	Range between maximum and minimum of combined axis accelerometer values
ACC_smv_low_ptp	Range between maximum and minimum of combined axis accelerometer low frequencies (0.3 to 2.5) values
ACC_smv_absmean	Absolute mean of combined axis accelerometer values
ACC_smv_low_absmean	Absolute mean of combined axis accelerometer low frequencies (0.3 to 2.5) values
ACC_smv_skew	Skew of combined axis accelerometer values
ACC_smv_low_skew	Skew of combined axis accelerometer low frequencies (0.3 to 2.5) values
ACC_smv_kurt	Kurtosis of combined axis accelerometer values
ACC_smv_low_kurt	Kurtosis of combined axis accelerometer low frequencies (0.3 to 2.5) values
ACC_smv_iqr	Inter quartile range of combined axis accelerometer values
ACC_smv_low_iqr	Inter quartile range of combined axis accelerometer low frequencies (0.3 to 2.5) values
ACC_smv_absarea	Absolute area of combined axis accelerometer values
ACC_smv_low_absarea	Absolute area of combined axis accelerometer low frequencies (0.3 to 2.5) values
ACC_smv_area	Area of combined axis accelerometer values
ACC_smv_low_area	Area of combined axis accelerometer low frequencies (0.3 to 2.5) values
ACC_smv_rmse_comb	Combination of the three RMS values for all three accelerometer axis
ACC_smv_rmse	RMS of the combined axis accelerometer value
ACC_smv_rms	Root mean square of combined axis accelerometer values
ACC_smv_low_rms	Root mean square of combined axis accelerometer low frequencies (0.3 to 2.5) values
ACC_smv_mean_crossings	Mean crossings of combined axis accelerometer values
ACC_smv_dom_freq	Dominant frequency of combined axis accelerometer values

ACC_smv_total_power	Sum of the total power of combined axis accelerometer values
ACC_smv_power_skew	Skew of the power of combined axis accelerometer values
ACC_smv_power_kurt	Kurtosis of the power of combined axis accelerometer values
ACC_smv_power_mean	Mean power of combined axis accelerometer values
ACC_smv_psd_0	First power spectral density component of combined axis accelerometer values
ACC_smv_psd_1	Second power spectral density component of combined axis accelerometer values
ACC_smv_psd_2	Third power spectral density component of combined axis accelerometer values
ACC_smv_psd_3	Fourth power spectral density component of combined axis accelerometer values
ACC_smv_psd_4	Fifth power spectral density component of combined axis accelerometer values
ACC_smv_psd_5	Sixth power spectral density component of combined axis accelerometer values
ACC_smv_psd_6	Seventh power spectral density component of combined axis accelerometer values
ACC_smv_psd_7	Eight power spectral density component of combined axis accelerometer values
ACC_smv_psd_8	Ninth power spectral density component of combined axis accelerometer values
ACC_smv_psd_9	Tenth power spectral density component of combined axis accelerometer values
ACC_smv_psd_10	Eleventh power spectral density component of combined axis accelerometer values
ACC_smv_psd_11	Twelfth power spectral density component of combined axis accelerometer values

ACC_smv_psd_12	Thirteenth power spectral density component of combined axis accelerometer values
ACC_smv_psd_13	Fourteenth power spectral density component of combined axis accelerometer values
ACC_smv_psd_14	Fifteenth power spectral density component of combined axis accelerometer values
ACC_smv_psd_15	Sixteenth power spectral density component of combined axis accelerometer values
ACC_smv_psd_16	Seventeenth power spectral density component of combined axis accelerometer values
ACC_smv_low_dom_freq	Dominant frequency of combined axis accelerometer low frequencies (0.3 to 2.5) values
ACC_smv_low_total_power	Sum of the total power of combined axis accelerometer low frequencies (0.3 to 2.5) values
ACC_smv_low_power_skew	Skew of the power of combined axis accelerometer low frequencies (0.3 to 2.5) values
ACC_smv_low_power_kurt	Kurtosis of the power of combined axis accelerometer low frequencies (0.3 to 2.5) values
ACC_smv_low_power_mean	Mean of the power of combined axis accelerometer low frequencies (0.3 to 2.5) values
ACC_smv_low_psd_0	First power spectral density component of combined axis accelerometer low frequencies (0.3 to 2.5) values
ACC_smv_low_psd_1	Second power spectral density component of combined axis accelerometer low frequencies (0.3 to 2.5) values
ACC_smv_low_psd_2	Third power spectral density component of combined axis accelerometer low frequencies (0.3 to 2.5) values
ACC_smv_low_psd_3	Fourth power spectral density component of combined axis accelerometer low frequencies (0.3 to 2.5) values
ACC_smv_low_psd_4	Fifth power spectral density component of combined axis accelerometer low frequencies (0.3 to 2.5) values

ACC_smv_low_psd_5	Sixth power spectral density component of combined axis accelerometer low frequencies (0.3 to 2.5) values
ACC_smv_low_psd_6	Seventh power spectral density component of combined axis accelerometer low frequencies (0.3 to 2.5) values
ACC_smv_low_psd_7	Eight power spectral density component of combined axis accelerometer low frequencies (0.3 to 2.5) values
ACC_smv_low_psd_8	Ninth power spectral density component of combined axis accelerometer low frequencies (0.3 to 2.5) values
ACC_smv_low_psd_9	Tenth power spectral density component of combined axis accelerometer low frequencies (0.3 to 2.5) values
ACC_smv_low_psd_10	Eleventh power spectral density component of combined axis accelerometer low frequencies (0.3 to 2.5) values
ACC_smv_low_psd_11	Twelfth power spectral density component of combined axis accelerometer low frequencies (0.3 to 2.5) values
ACC_smv_low_psd_12	Thirteenth power spectral density component of combined axis accelerometer low frequencies (0.3 to 2.5) values
ACC_smv_low_psd_13	Fourteenth power spectral density component of combined axis accelerometer low frequencies (0.3 to 2.5) values
ACC_smv_low_psd_14	Fifteenth power spectral density component of combined axis accelerometer low frequencies (0.3 to 2.5) values
ACC_smv_low_psd_15	Sixteenth power spectral density component of combined axis accelerometer low frequencies (0.3 to 2.5) values
ACC_smv_low_psd_16	Seventeenth power spectral density component of combined axis accelerometer low frequencies (0.3 to 2.5) values
pitch_mean	Mean of pitch value
pitch_std	Standard deviation of pitch value
pitch_max	Maximum of pitch value
pitch_min	Minimum of pitch value
roll_mean	Mean of roll value
roll_std	Standard deviation of roll value
roll_max	Maximum of roll value
roll_min	Minimum of roll value

TEMP_mean	Mean of skin temperature values
TEMP_median	Median of skin temperature values
TEMP_min	Minimum of skin temperature values
TEMP_max	Maximum of skin temperature values
TEMP_std	Standard deviation of skin temperature values
TEMP_var	Variance of skin temperature values
TEMP_ptp	Range between maximum and minimum of skin temperature values
TEMP_absmean	Absolute mean of skin temperature values
TEMP_skew	Skew of skin temperature values
TEMP_kurt	Kurtosis of skin temperature values
TEMP_iqr	Inter quartile range of skin temperature values
TEMP_absarea	Absolute area of skin temperature values
TEMP_area	Area of skin temperature values
TEMP_rms	Root mean square of skin temperature values
TEMP_mean_crossings	Mean crossings of skin temperature values
